# Quantitative analysis of three-dimensional morphology and membrane dynamics of red blood cells during temperature elevation

**DOI:** 10.1038/s41598-019-50640-z

**Published:** 2019-10-01

**Authors:** Keyvan Jaferzadeh, MinWoo Sim, NamGon Kim, InKyu Moon

**Affiliations:** Department of Robotics Engineering, DGIST, 333 Techno Jungang-daero, Hyeonpung-myeon, Dalseong-gun, Daegu, 42988 South Korea

**Keywords:** Nanoscale biophysics, Image processing, Preclinical research, Biophotonics

## Abstract

The optimal functionality of red blood cells is closely associated with the surrounding environment. This study was undertaken to analyze the changes in membrane profile, mean corpuscular hemoglobin (MCH), and cell membrane fluctuations (CMF) of healthy red blood cells (RBC) at varying temperatures. The temperature was elevated from 17 °C to 41 °C within a duration of less than one hour, and the holograms were recorded by an off-axis configuration. After hologram reconstruction, we extracted single RBCs and evaluated their morphologically related features (projected surface area and sphericity coefficient), MCH, and CMF. We observed that elevating the temperature results in changes in the three-dimensional (3D) profile. Since CMF amplitude is highly correlated to the bending curvature of RBC membrane, temperature-induced shape changes can alter CMF’s map and amplitude; mainly larger fluctuations appear on dimple area at a higher temperature. Regardless of the shape changes, no alterations in MCH were seen with temperature variation.

## Introduction

Mature red blood cells (RBCs) or the so-called erythrocytes are the main cell types present in blood circulation. The main role of RBCs is to deliver oxygen and nutrients to tissues, and transfer carbon dioxide from tissues to lung to be exhaled out from the system. In order to survive their 120 days lifetime, RBCs adapt to their surroundings by subtle regulation of membrane organization and metabolism. RBCs are different from most other cells in that they lack intracellular organelles such as the nucleus, mitochondria, or endoplasmic reticulum. This allows RBCs to have an increased capacity to transport oxygen and absorb dioxide carbon. The biconcave shape of RBCs is optimal for maximal deformation, maximum surface at a given volume, rapid changes, and survival of the cell during its passages through the micro capillary system. This is possible due to the absence of a three-dimensional cytoskeleton in RBCs. Investigating the shape changes of erythrocytes induced by various conditions have interested researchers for a long time. Particularly, temperature plays ubiquitous roles in steady-state volume change, ion exchange rate, hemolysis rate, membrane dynamics and deformation of cells^1–5^. Several proposals suggest that RBC membrane become less stable when they are exposed to the temperatures above the normal body temperature. One reason is that the dynamics of cell surface membranes are determined by the fluid state of the lipid bilayer which, in turn, is affected by temperature^6^. The second proposal suggests that at the higher temperature membrane phospholipids start to melt and can cause RBC to rupture. Another study showed that the unilamellar state of RBC membrane is stable at the temperatures ~37 °C but at the higher temperature, it is changed to a multibilayer^7^. Cell membrane fluctuations (CMF) of the elastic membrane relies on the hypothesis that the driving force of fluctuations is purely thermal. Therefore, thermal changes can directly change membrane flickering since it is thermal dependent. Thermal-induced changes on membrane profile can also affect RBC’s CMFs map and amplitude. It is shown that bending curvature of the RBC membrane is the main contribution of membrane fluctuations map and its magnitude^8,9^.

In this study, we monitored the changes in the shape and CMF map of RBCs with respect to varying temperatures at the single-RBC level in a label-free manner. The three-dimensional (3D) images are provided by digital holography (DH) in microscopic configuration. It can record the phase retardation when the coherent light source travels through microscopic objects. Phase retardation is related to two factors, namely, cell thickness and the intracellular refractive index, a property linked to the water and protein content of the cells^10^. Also, DH provides quantitative phase images (QPIs) at the single-cell level with nanometer accuracy. Since this method records phase changes instead of amplitude, staining by a specific dye is not required. This makes DH a well-suited technique for label-free studying. DH in microscopic configuration has been utilized in studies of various types of cells^11–19^ and also human red blood cells^20–26^. Parameters such as RBC volume, surface area, sphericity index, refractive index and RBC membrane fluctuations are essential parameters that can be evaluated using DH.

The main motivation of this study with the previously published works is that unlike the previous work, we are very much interested in the single-RBC level changes. We are able to monitor and track changes very precisely at the single cell level with the digital holography at the microscopic level. QPI is related to two factors of cell thickness and intracellular material of the RBC. Therefore, any changes in either thickness or intracellular during temperature changes can be monitored. We believe that thermal-induced change imbalances equilibrium of the RBC membrane in different ways and it can be monitored by DH.

### Label-free digital holographic imaging

**Figure 1:**
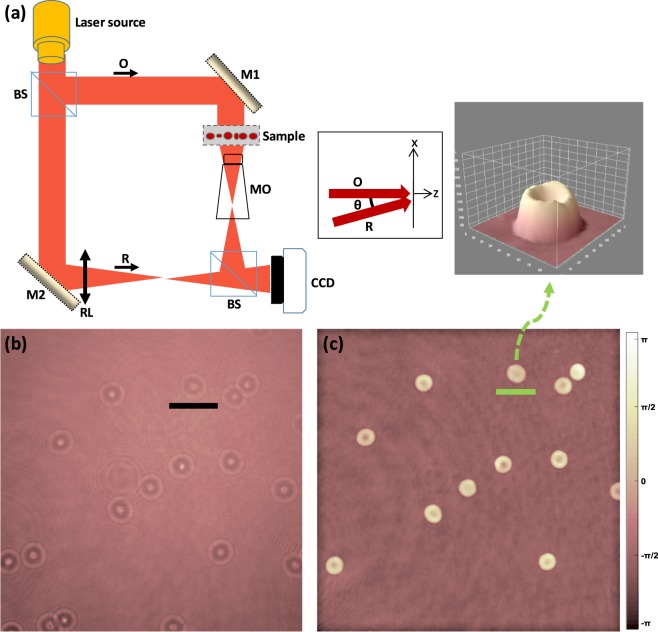
(**a**) Schematic diagram of a digital holographic in microscopic configuration; inset shows the off-axis configuration, (**b**) recorded hologram (black bar is 500 µm), and (**c**) reconstructed phase image (green bar is 10 µm); inset is the single cell 3D representation.

The general layout of an off-axis digital holographic microscope (DHM) is presented in Fig. 1. The hologram recording is based on the Mach-Zender interferometer. The coherent laser source is divided into object (*O*) and reference beams (*R*) by using a beam splitter (Fig. 1(a)). The object beam illuminates the specimen, and a microscope objective (MO) collects and magnifies the object wave front. The object and reference wave fronts are joined by a beam collector to create the hologram with a small tilt angle between them to provide the “Off-axis” geometry. At the end point, interferograms (Fig. 1(b)) are recorded by a charge-coupled-device (CCD) camera, and the data is transmitted to a personal computer for numerical reconstruction.

The numerical reconstruction is explained in detail in references^27,28^. Briefly, the interference between the object wave *O* and reference wave *R* constructs the recorded hologram *I*_*H*_ by the following equation:

1 $${I}_{H}={|R|}^{2}+{|O|}^{2}+{R}^{\ast }O+{O}^{\ast }R,$$

where R^*^ and O^*^ denote the complex conjugates of the reference and object beams, respectively. The small tilt angle between *O* and *R* enables us to eliminate the parasitic orders and isolate the real image from twin image and zero-order noise. In order to enable this, an appropriately sized spatial filter is implemented to cover only the bandwidth of the real image. By Fourier transforming the off-axis hologram followed by its multiplication (in frequency-domain) by the filter and applying inverse Fourier transform, only the real image information is preserved by:

2 $${I}_{H}^{F}=IFFT\{FFT({I}_{H})\times Filter\}={R}^{\ast }O,$$

where *FFT* is the fast Fourier transform and *IFFT* is the inverse of *FFT*. To reconstruct the phase image, the filtered hologram (*I*_*H*_^*F*^) is multiplied by the digital planer reference wave *R*_*D*_:

3 $${R}_{D}(k,l)=\exp [i(\frac{2\pi }{\lambda })({k}_{x}k\Delta x+{k}_{y}k\Delta y)],$$

where *λ* is the wavelength of the laser source, Δ*x* and Δy are the sampling intervals in the image plane, and *k*_*x*_ and *k*_*y*_ are the wave vectors. The image reconstruction is expressed by the Fresnel approximation, as follows:

4 $$\begin{array}{l}\varPsi (m,n)=A\Phi (m,n)\exp [\frac{i\pi }{\lambda d}({m}^{2}\Delta {\xi }^{2}+{n}^{2}\Delta {\eta }^{2})]\\ \times FFT{\{{R}_{D}(k,l){I}_{H}^{F}(k,l))\times \exp [\frac{i\pi }{\lambda d}({k}^{2}\Delta {x}^{2}+{l}^{2}\Delta {y}^{2})]\}}_{m,n},\end{array}$$

where *A* is a complex constant value, *k*, *l*, *m*, and *n* are integers (*−N*/2 ≤ *k*, *l*, *m*, *n* ≤ *N*/2; and *N* × *N* is the number of pixels in the CCD camera 1024 × 1024), and Φ(*m*,*n*) is the digital phase mask calculated by:

5 $$\Phi (m,n)=\exp [\frac{-i\pi }{\lambda D}({m}^{2}\Delta {\xi }^{2}+{n}^{2}\Delta {\eta }^{2})],$$

where Δ*ξ* and Δ*η* are the sampling intervals in the observation plane expressed by:

6 $$\Delta \xi =\Delta \eta =\frac{\lambda d}{N\Delta x},$$

where *d* denotes distance between camera plane and observation plane. A fine adjustment of *k*_*x*_ and *k*_*y*_ can be performed in the absence of fringes by removal of residual gradients or curvature of the reconstructed phase distribution in some areas of the image where a constant phase is presumed. *D* is the parameter that must be adjusted to compensate the wave-front curvature according to the distance between MO and specimen, and between MO and the image plane. The digital phase mask can resolve the phase aberrations caused by inserting a microscopic objective in the object wave arm, as shown in Fig. 1(a). Eventually, the phase image (Fig. 1(c)) can be obtained by the argument of:

7 $$\varphi (x,y)={\tan }^{-1}\{\frac{\text{Im}[\Psi (m,n)]}{\mathrm{Re}[\Psi (m,n)]}\},$$

Since the phase values are limited between −*π* and +*π*, the result is given modulo 2*π*; discontinuities with values near to 2*π* might appear in non-flat large samples (we did not see phase jump in our RBCs). The quantitative phase image can be represented in the form of optical path difference (OPD) by:

8 $$OPD(x,y)=\frac{\lambda \times \varphi (x,y)}{2\pi },$$

The OPD is related to two factors of cell thickness and the intracellular refractive index, a property linked to the protein and water content of cells. Images were acquired by off-axis DHM configuration on a commercially available DHM T-1001 procured from LynceeTec SA (Lausanne, Switzerland) equipped with a motorized *x*-*y* stage (Märzhäuser Wetzlar GmbH & Co. KG, Wetzlar, Germany, ref. S429). After recording the hologram, the images were reconstructed with a standard PC at the rate of 3 QPI images per second. For the cell membrane dynamic analysis, the sampling rate was set at 10 Hz and the sampling time was 100 seconds. We used two “MO”s of 63×/1.3NA (field of view = 92 μm) and 40×/0.75NA (field of view = 150 μm) in our configuration. The laser source (666 nm) delivered an intensity of ~200 μW/cm^2^ to the specimen plane with an exposure time of approximately 0.4 ms. Image reconstruction and all analyses were carried out using the MATLAB software.

## Methods

### Red blood cell sample preparations

Approximately 5 ml blood was collected from three healthy male donors using a syringe. It was diluted at a ratio of 1:10 (v/v) in cold PBS buffer (pH 7.4, 138 mM NaCl, 27 mM KCl, 10 mM Na_2_HPO_4_, and 1 mL KH_2_PO_4_). Blood cells were sedimented by centrifuging at 200 g, 4 °C for 10 min, following which the buffy coat was gently collected, and washed once in PBS buffer for 2 min at 4 °C. Finally, the isolated erythrocytes were suspended in HEPA buffer (280 mOsm, 15 mM HEPES pH 7.4, 130 mM NaCl, 5.4 mM KCl, 10 mM glucose, 1 mM CaCl_2,_ 0.5 mM MgCl_2_ and 1 mg/ ml bovine serum albumin) at 0.2% hematocrit; 1 ml of the erythrocyte suspension was diluted to 15 ml using HEPA buffer. Two experimental conditions were considered. For the first experiment, ~40 µl of the final erythrocyte suspension was introduced into the imaging slide consisting of two coverslips. The bottom coverslip was coated with polyornithine to ensure that cells adhere to the coverslip surface. For the second experiment, 200 µl of the final erythrocyte suspension was dropped on the 18 mm imaging slide of a round chamber. The glass and chamber were mounted on the DHM device and incubated for 10 minutes at 17 °C under conditions of 5% CO_2_ and high humidity (Chamlide WP incubator system, LCI, Seoul, South Korea). This ensures that the cells adhere well to the glass. For the RBC membrane fluctuation analysis, the RBCs were imaged at 17 °C, 23 °C, 37 °C and 41 °C, with the sensitivity of ±0.1 °C. The RBCs were imaged continually for all other parameters. Only cells with the discocyte morphology (*n* > = 36) are considered for the final analysis; all other morphologies are excluded from the sample set.

All the blood samples in this study were obtained with informed consent from all subjects. All procedures were performed in accordance with the internal protocols of our laboratory, which is in accordance with the guidelines and regulations (DGIST-180713-BR-012-01) approved by the bio-safety committee, DGIST University, Korea and institutional review boards (IRBs) in Korea. The experiments were finished within a few hours after sample collection.

## Results and Discussions

### Biochemical and morphological parameters

Several parameters related to the morphological aspects, RBC membrane fluctuations and clinically relevant at the single-RBC level, were analyzed. Before analysis, several single RBCs (only RBCs with the perfect biconcave morphology) were manually extracted from the original QPI images (see Fig. 1(c) and the inset). Single cells were then binarized to provide background and cell region masks. Applying the morphological operation of dilation to the background mask isolated it from the cell region. The first morphological-related variable considered was the projected surface area (PSA), defined as:

9 $$PSA=N{p}^{2},$$

where *N* is the total number of pixels within the RBC projected area resulting from the image binarization. Mean corpuscular hemoglobin (MCH) content is a clinically relevant parameter that can be evaluated by interferometric methods like DHM. Phase retardation is proportional to the protein component of the cell and, in case of RBCs, is mostly composed of hemoglobin. The refractive index is closely related to other properties of the red cell (volume, size, water content, temperature changes) and depends considerably on the cell environment. The individual dry weight of a red cell, however, is a property which remains more or less unchanged when it has reached a final value during the maturation process of the reticulocyte:

10 $$MCH=\frac{10\times \overline{OPD}\times (PSA)}{\lambda {\alpha }_{HB}},$$

where $\overline{OPD}$ is the average optical path difference (OPD) over the projected surface area of the cell (see Eq. 11), *λ* is the wavelength of the light source of the setup, and *α*_HB_ = 0.00196 *dl*/*g* is the specific refraction increment related mainly to the protein concentration of hemoglobin.

11 $$\overline{OPD}=\sum _{(i,j)\in {S}_{p}}OPD(i,j),$$

where the summation achieved considering all the pixels (*i*, *j*) belongs to the projected surface *S*_*p*_ of the RBC, and *OPD*(*i*, *j*) is the OPD value at pixel (*i*, *j*). Another morphological property is the sphericity coefficient *k*, which is expressed as the ratio of the central RBC OPD value (*OPD*_*c*_) to the OPD value at half of its radius (*OPD*_*r*_):

12 $$k=\frac{OP{D}_{c}}{OP{D}_{r}},$$

RBC membrane fluctuations were also measured during the period of temperature changes. For this analysis, four temperatures were considered: 17 °C, 23 °C, 37 °C and 41 °C. At each temperature, we recorded 100 holograms having sampling rate of 10 Hz. Holograms were numerically reconstructed after the experiment. The model for measuring the RBC membrane fluctuations is presented in the following references^23,24^. Simply put, a region of interest (ROI) with two independent variables are required; $std(OP{D}_{cell}+OP{D}_{bkgd})(x,y)$ which is the temporal deviation within the RBC area (combining both the cell fluctuations and noise), and $std(OP{D}_{bkgd})$ which is the mean of the temporal deviation of all the pixels outside the RBC area. Accordingly, the fluctuations for each single pixel *CMF*(*x*, *y*) is evaluated by the formula:

13 $$CM{F}_{RBC}(x,y)=\sqrt{{(std(OP{D}_{cell}+OP{D}_{bkgd})(x,y))}^{2}-{(std(OP{D}_{bkgd}))}^{2}},$$

## Discussions

### RBC trapped between cover slip and glass

**Figure 2:**
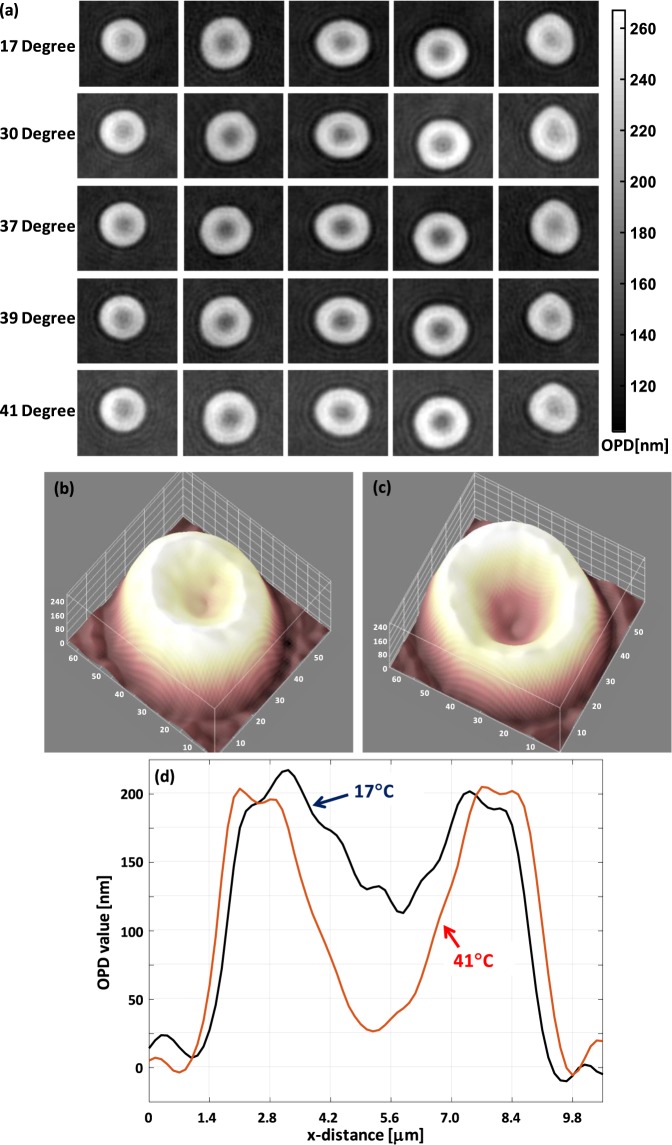
(**a**) Gallery of RBC images; same RBCs are shown at different temperatures, (**b**) RBC at 17 °C, (**c**) the same RBC at 41 °C, and (**d**) cross-section of (**b**,**c**) drawn together. PSA for the RBC in (**b**,**c**), respectively, are 60 µm^2^ and 64 µm^2^. Sphericity coefficient for the RBC in (**b**,**c**), respectively, are 0.65 and 0.45. MCH = 32.3 pg and 32.5 pg for RBC shown in (**b**,**c**), respectively.

Figure 2 shows RBC images at different temperature and the profile and cross-section of one RBC at two temperatures, 17 °C and 41 °C. This is the case when cells are trapped between cover slip and glass. As is clearly observed, the dimple section (central portion) of the RBC differs at both temperature parameters.

**Figure 3:**
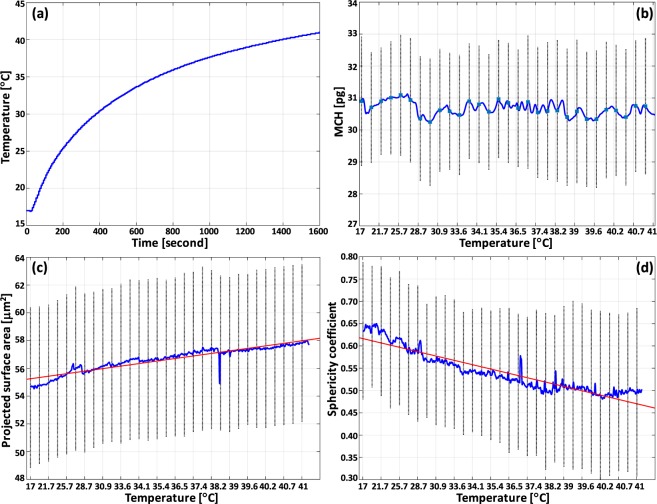
(**a**) Rising temperature duration, (**b**) MCH (**c**), projected surface area, and (**d**) sphericity coefficient changes versus temperature elevation (*n* > = 36 cells). F-statistics performed on data shown in (**c**,**d**) suggests that the slope of the linear regression line is significantly different from zero; p-value < 0.05. Error bars shown in the plots represent twice the corresponding standard deviations.

The impact of increasing temperature was assessed by measuring the morphological parameters, MCH and magnitude of fluctuation rates. Figure 3(a) shows the time course of temperature elevation for this study, wherein we observe that MCH remains unchanged (Fig. 3(b)), and showing only minor fluctuations around its average value (30.76 pg). Increasing temperatures result in an increase in PSA values and decrease in sphericity coefficient (see Fig. 3(c,d)). The results together most likely suggest that RBCs are losing the intracellular fluid and consequently the volume of RBC drops. At the beginning of the experiment the cell and extracellular medium are in the isotonic condition thus no movement of water has occurred, and RBCs can preserve their shape. After some moments of increasing temperature, the surrounding medium gradually starts to evaporate, causing imbalance between intracellular and extracellular fluid. Also, intracellular fluidity rises since it is directly affected by the higher temperature. Therefore, a gradient of concentration between RBC membrane and the extracellular medium is created, and water molecules start leaving RBC to diminish the gradient. This causes RBC to lose water and consequently, volume decreases. Also, this causes the concentration of the materials inside the RBC increases comparing with the lower temperature condition. This is due to that there is less water inside the RBC at the higher temperature. We are not entirely able to confirm the volume loss since the refractive index of RBC is required to convert OPD value to actual RBC thickens and then calculate the volume. The RBC’s refractive index is obtained at the room temperature, but it varies at different temperature. Additionally, if there is a change in concentrations of materials within RBC, it indeed alters integral refractive index of RBC.

**Figure 4:**
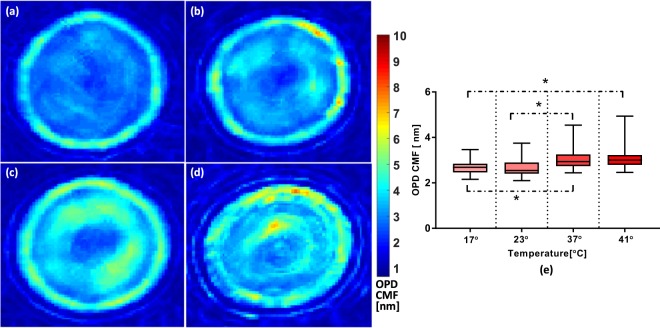
Fluctuations map for an RBC at different temperatures. (**a**) 17 °C, (**b**) 23 °C, (**c**) 37 °C, (**d**) 41 °C, (**e**) box plot representation of amplitude of CMF for the four different temperatures. An asterisk *indicates that results are significantly different according to the two-sample Kolmogorov-Smirnov test; p-value < 0.05.

Figure 4 demonstrates the CMF maps for an RBC measured at the four different temperatures. The deviations map of the cell is evaluated by Eq. (13). The *std*(*OPD*_*cell*_ + *OPD*_*bkgd*_)(*x*,*y*), which is the temporal deviation within the ROI combining both the cell fluctuations and noise, is measured. Furthermore, we also calculate the average value of *std*(*OPD*_*bkgd*_), which is the temporal deviation of all the pixels outside the RBC’s projected area. The two measured values are substituted into Eq. (13) and the map is evaluated. CMF amplitude of the whole cell is the average of the CMF(*x*,*y*) over the projected area of the whole cell. The CMF amplitude for each temperature is also calculated, and the comparison is presented as a box plot (Fig. 4(e)). It is clearly observed in Fig. 4 that the CMF map at lower temperatures is dominant at the ring section of the membrane. It is shown by Popescu that RBC membrane fluctuations have direct relation with the Gaussian curvature of RBC^8^. The deformation of membrane is dominant at the area in which the Gaussian curvature approaches zero. Higher temperature caused losing intracellular fluid, which in turn resulted in an RBC with less sphericity index. Thus, the Gaussian curvature is modified and therefore, fluctuations map differs for the case of higher temperature. We also found out in our previous work that there is a significant negative correlation between CMF value and sphericity coefficient^24^. Also, the Kolmogorov-Smirnov test shows that the ring’s CMF is greater than the corresponding value for the dimple section for all temperatures (data not shown here).

It is worth mentioning that no changes in morphology, shape, and CMF are observed when the cells are left in room temperature for a time course of one hour (data is not shown here).

### RBCs imaged on chamber

**Figure 5:**
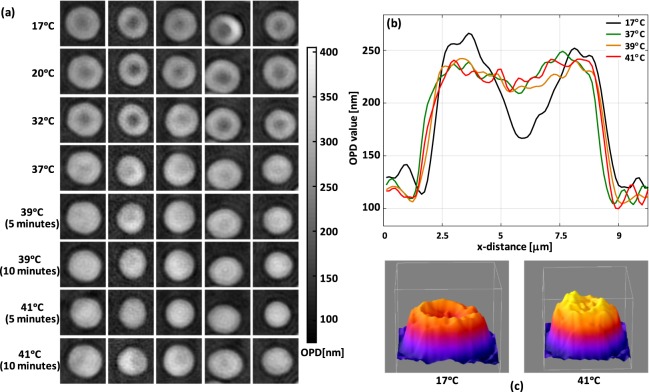
(**a**) Gallery of RBC images; same RBCs are shown at different temperatures, (**b**) cross-section of the same RBC shown at different temperature (**c**) 3D representation of the RBC in 17 °C and 41 °C. PSA for the RBC in (**b**,**c**), respectively, are 49 µm^2^ and 50 µm^2^. Sphericity coefficient for the RBC in (**b**,**c**), respectively, are 0.86 and 0.91. MCH = 30.5 pg and 30.9 pg for RBC shown in (**b**) and (**c**), respectively.

In the second experiment 200 µl of the suspension was dropped on imaging glass of 18 mm round coverslip chamber. The temperature was elevated and at each temperature, RBCs were imaged. RBCs were imaged 5 and 10 minutes after that temperature reached to the desired level. Figure 5 shows a gallery of RBC images at different temperature and a profile of one RBC at different temperature.

**Figure 6:**
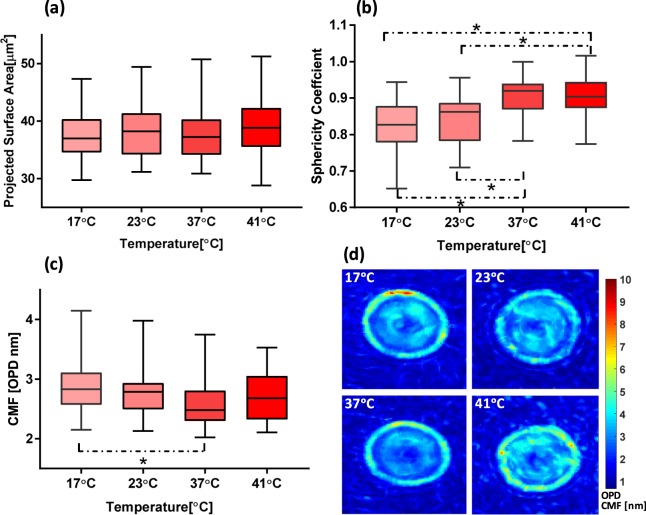
(**a**) Projected surface area, (**b**) sphericity coefficient and (**c**) CMF value at different temperatures. (**d**) represents CMF map at different temperatures. An asterisk *indicates that results are significantly different according to the two-sample Kolmogorov-Smirnov test; p-value < 0.05; n > = 36 cells).

Figure 6 shows the effect of temperature elevation on the shape and membrane fluctuations. It can be readily seen that the RBCs sphericity coefficient at a lower temperature is less than the same value at a higher temperature. No significant change in PSA is observed but CMF value for two temperature of 17 °C and 37 °C are significantly different (p-value < 0.005). The mean corpuscular volume of the samples was measured with a Sysmex XP-300 Impendence volume analyzer, a commonly used in hematology laboratories to obtain complete blood count. No changes in volume or MCH is found at different temperatures (data not shown here). We did not observe significant reversible changes in the membrane of RBC and cells preserved their shape. It is worth mentioning that some RBCs start to gradually lose their membrane stability. Accordingly, RBCs with spiculated shape started to appear.

The cell membrane is made of phospholipids which are rather fluid. The phospholipids are more rigid at the lower temperature and it becomes soften at the higher temperature. More specifically, the fatty acid tails of the phospholipids become less rigid and allow more movement of proteins and other molecules in and through the membrane. At some temperature above the normal ambient level, the membrane is more unstable and very fluid. Another hypothesis is that the transformation of the membrane bilayer occurs when the normal ambient temperature of the cell is exceeded. Accordingly, the growth temperature of the cell is a critical point where optimal membrane bilayer stability occurs.

## Conclusions

The mechanical properties and shape of cells might be modified by temperature variations. In this paper, we analyzed the effect of temperature on several parameters of RBCs by using digital holography in the microscopic configuration. Temperatures were elevated during a time course of less than one hour. Our results indicate that RBCs retain their normal morphology, although there are changes in some parameters related to the profile of RBC. Sphericity coefficient changes when cells are induced to the temperature. Another hypothesis is that temperature enhances the fluidity of some membrane compounds.

## Data Availability

The datasets used and/or analyzed during the current study are available from the corresponding author on reasonable request.
